# P-941. Short- and Long-term Mortality of Infective Endocarditis in the Veteran Population

**DOI:** 10.1093/ofid/ofae631.1132

**Published:** 2025-01-29

**Authors:** Matthew Davis, Kari A Mergenhagen, Arthur Chan, Jiachen Xu, Bethany A Wattengel, Ashley O’Leary

**Affiliations:** VA WNY Healthcare System, Buffalo, New York; VA WNY Healthcare System, Buffalo, New York; Veterans Affairs Western New York Healthcare System, Buffalo, New York; Veterans Affairs Western New York Healthcare System, Buffalo, New York; VA WNY Healthcare System, Buffalo, New York; VA WNY Healthcare System, Buffalo, New York

## Abstract

**Background:**

The aim of this study was to evaluate the short- and long-term mortality of infective endocarditis within the veteran population.

**Figure:**
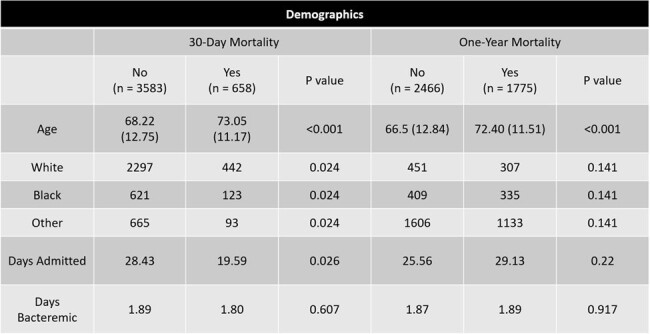

**Methods:**

This study was a retrospective, national study of veterans > 18 years old between 1/1/2010-3/1/2023 diagnosed with endocarditis. Endocarditis was defined by ICD coding, patients having at least one positive blood culture and having an echocardiogram performed. during admission. The primary objective was to evaluate the rate of 30-day and one-year mortality of the study population.

Secondary endpoints looked at correlation between mortality and other factors such as comorbidities, causative organism, drug/alcohol disorders, smoking status, septic emboli, fever, and the use cardio-protective medications.

Charlson Comorbidity Scores

**Figure:**
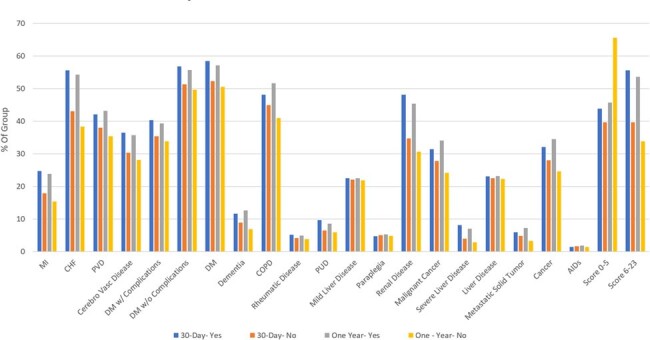

**Results:**

4,241 hospitalized patients who received care at a Veterans Affairs Hospital and were diagnosed with bacterial endocarditis during the study timeframe were included. The primary outcome of short- and long-term mortality was seen in 658/4,241 patients (15.5%) and 1775/4,241 patients (41.9%) respectively.

Enterococcus faecalis endocarditis was the most prevalent organism (17.2% of infections). MRSA endocarditis was associated with a clinically significant increase in mortality at both 30-days and one-year (p < 0.001). The diagnosis of septic emboli showed a statistically significant increase in short-term mortality with 106/427 patients diagnosed experiencing mortality within 30-days (p < 0.001) Fever during admission showed a statistically significant increase in mortality for both short- and long-term mortality rates (p < 0.001).

**Figure:**
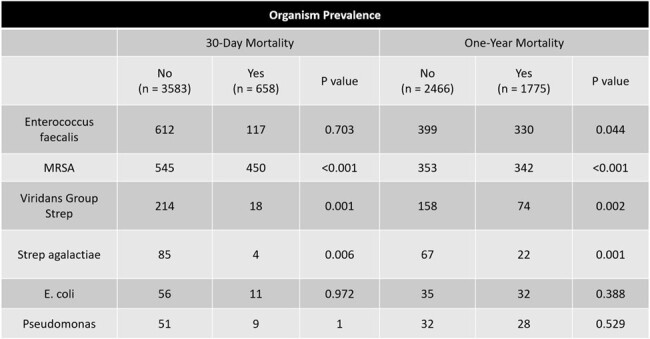

**Conclusion:**

Mortality within one-year was found to be 41.9% amongst our patient population. Identifying possible risk factors for increased mortality in this population may assist in decreasing both the short- and long-term mortality of those with infective endocarditis. Future directions for research could include assessing cardio-protective medications in this population on a larger scale to assess potential benefit.

**Figure:**
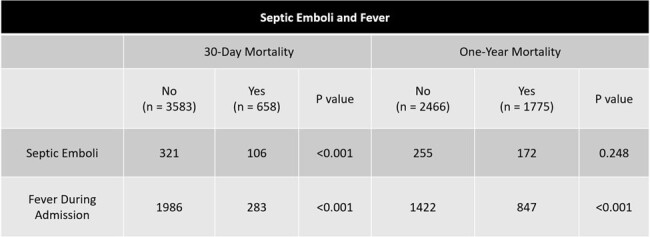

**Disclosures:**

**All Authors**: No reported disclosures

